# Whole-Genome Sequences of Human Monkeypox Virus Strains from Two 2022 Global Outbreak Cases in Western New York State

**DOI:** 10.1128/mra.00846-22

**Published:** 2022-11-14

**Authors:** Mondraya Howard, Joel J. Maki, Sara Connelly, Dwight J. Hardy, Andrew Cameron

**Affiliations:** a Department of Pathology and Laboratory Medicine, University of Rochester Medical Center, Rochester, New York, USA; b Department of Microbiology and Immunology, University of Rochester Medical Center, Rochester, New York, USA; Portland State University

## Abstract

The genomes of two human monkeypox virus strains from recently reported cases in our local region that were associated with the 2022 global outbreak were sequenced. Genomes from clinical isolates provide valuable information for epidemiological tracking and analysis of strain evolution and can be especially important during the early phases of outbreaks.

## ANNOUNCEMENT

Monkeypox is a disease acquired through infection with monkeypox virus (MPXV), an *Orthopoxvirus* species belonging to the *Poxviridae* family (1). Monkeypox classically presents with fever, swollen lymph nodes, and maculopapular rash involving the palms and soles (2). Previously, MPXV transmission had been largely zoonotic, with sporadic outbreaks reported in West and Central Africa (3–5). Cases outside this region were associated with travel or importation of animals; sustained human-to-human transmission was uncommon (6). In May 2022, monkeypox cases were identified in the United Kingdom and quickly spread to other countries (7). Initial analyses demonstrated that MPXV outbreak isolates arose from the West African clade, which is considered less lethal than the Congo Basin clade of MPXV (8). In July 2022, the World Health Organization declared monkeypox a global health emergency (9).

We report whole-genome sequences for the MPXV strains URMC_2207A101 and URMC_2207A102, which were isolated from genital lesion swab samples from two unique patients in Monroe County, New York, in July 2022. All specimens had tested positive (threshold cycle [*C_T_*] value, ~19) for MPXV nucleic acid using primers and probes identical to the Centers for Disease Control and Prevention MPXV and nonvariola orthopoxvirus generic real-time PCR tests (10). Total nucleic acid was extracted using the EZ1 DSP virus kit (Qiagen). DNA libraries (4 nM) were prepared (Illumina DNA preparation kit) and quantified (QuantiFluor One double-stranded DNA [dsDNA] system; Promega) for paired-end sequencing (MiSeq reagent kit v3 [2 × 75 bp]) on an Illumina MiSeq instrument.

**FIG 1:**
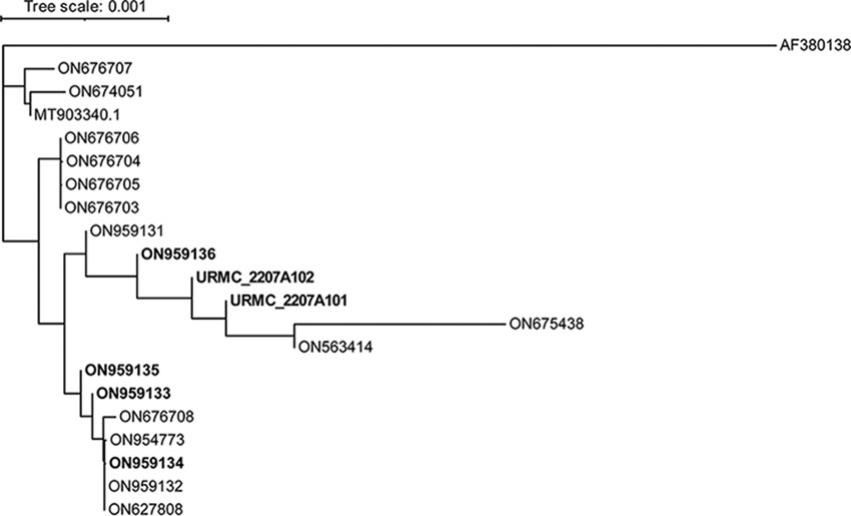
Phylogeny of MPXV genomes from the United States. A phylogeny was constructed using a Tamura-Nei distance model and neighbor-joining methodology in Geneious Prime v2022.1.1. GenBank accession number AF380138 (Zaire-96-I-16) was used as an outgroup for URMC_2207A101, URMC_2207A102, and other complete human MPXV genomes sequenced after December 2021. Genomes from New York State are bolded. The scale bar indicates substitutions per site.

Reads were assessed with a public Galaxy server (https://usegalaxy.eu) using variation analysis and consensus construction workflows established by the GalaxyProject MPXV analysis effort (https://galaxyproject.org/projects/mpxv) (11). Reads were processed with fastp (12) and mapped to MPXV-M5312_HM12_Rivers (GenBank accession number MT903340.1) using BWA-MEM v0.7.17; SnpEff-identified variants were used to build consensus whole-genome sequences (Table 1). Default workflow parameters were used; the minimum allelic frequency for consensus variants was 0.75, and sites with <5 reads were masked. Sequences were aligned in Geneious Prime v2022.1.1 using Mauve v1.1.3 (13). A phylogeny (Fig. 1), which included URMC_2207A101, URMC_2207A102, all complete U.S. MPXV genomes since 2021 in the Bacterial and Viral Bioinformatics Resource Center (www.bv-brc.org), GenBank accession number MT903340.1 (4), and GenBank accession number AF380138 (Zaire-96-I-16), was constructed using a Tamura-Nei distance model and neighbor-joining methodology in Geneious Prime (14). The consensus tree was visualized with iTOL (15). The average nucleotide identity (ANI) with respect to GenBank accession number MT903340.1 was determined with JSpeciesWS (16). Human reads were filtered prior to submission to the Sequence Read Archive (SRA). Briefly, BWA-MEM was used to map trimmed reads (Trimmomatic v0.38) against the GRCh38 reference human genome; nonhuman reads/identifiers were extracted from the BAM data with SAMtools v1.15.1, and then Seqtk v1.3 was used with the nonhuman identifiers to filter out human reads from the original fastq files.

**TABLE 1:** Sequencing metrics and accession numbers for sequenced MPXV genomes

Strain	Genome length (bp)	No. of reads mapped to reference^*a*^	Avg coverage (×)	GC content (%)	ANI (%) vs GenBank accession no.		GISAID accession no.	GenBank accession no.	SRA accession no.	Nucleotide substitutions	Amino acid substitutions
					MT903340.1	AF380138					
URMC_2207A101	197,209	1,394,110	537	33.10	99.96	99.07	EPI_ISL_14003930	OP588945	SRR21871733	G55133A, C64426T, C170673T, C185767T, G190660A	NBT03_gp174:R84K, OPG074:R665C, OPG210:S1471F
URMC_2207A102	197,209	434,597	167	33.00	99.96	99.07	EPI_ISL_14251112	OP588946	SRR21871732	G55133A, C64426T, C89698T, C170673T, G190660A	NBT03_gp174:R84K, OPG074:R665C, OPG110:H23Y

*^a^* Relative to MPXV-M5312_HM12_Rivers (GenBank accession number MT903340.1).

The two sequences were B.1.3 lineage, clade IIb (Nextclade v2.6.1), consistent with other sequences from the 2022 MPXV outbreak, descendants of the A.1.1 lineage exported from Nigeria in 2021 (17). In total, 72 nucleotide substitutions, resulting in 32 amino acid changes in 25 different genes, were observed in URMC_2207A101 and URMC_2207A102, compared to GenBank accession number MT903340.1. Four nucleotide substitutions, i.e., G55133A (OPG074:R665C), C64426T (no affected codon), C170673T (no affected codon), and G190660A (NBT03_gp174:R84K), were common to URMC_2207A101 and URMC_2207A102. Additionally, C89698T (OPG110:H23Y) in URMC_2207A102 and C185767T (OPG210:S1471F) in URMC_2207A101 were observed. URMC_2207A101 and URMC_2207A102 formed a monophyletic clade with MPXV strains from Virginia and Massachusetts (GenBank accession numbers ON675438 and ON563414, respectively) (Fig. 1).

These data confirmed the expected lineage for our local MPXV outbreak cluster. Sequencing of viral genomes from clinical specimens provides important information regarding regional trends and mutations impacting diagnostic tests and aids in epidemiological investigations at the local and national levels during large outbreaks.

This study was performed under University of Rochester Medical Center (URMC) institutional review board-approved protocol RSRB00007269.

### Data availability.

Genome sequences have been deposited in GISAID (accession numbers EPI_ISL_14003930 and EPI_ISL_14251112) and GenBank (accession numbers OP588945 and OP588946). Processed reads that mapped to MT903340.1 are available in the NCBI SRA under accession numbers SRR21190141 and SRR21190140. Reads with human reads filtered are available in the SRA under accession numbers SRR21871733 and SRR21871732.
